# A new species of *Dictyotenguna* Song & Liang, 2012 from China (Hemiptera, Fulgoromorpha, Dictyopharidae)

**DOI:** 10.3897/zookeys.429.6950

**Published:** 2014-07-29

**Authors:** Yan-Li Zheng, Xiang-Sheng Chen

**Affiliations:** 1 Institute of Entomology, Guizhou University; The Provincial Key Laboratory for Agricultural Pest Management of Mountainous Region, Guiyang, Guizhou 550025 P.R. China; 2 Guizhou Normal College, Guiyang, Guizhou, China 55001 P.R. China

**Keywords:** Fulgoroidea, Oriental Region, morphology, taxonomy, distribution

## Abstract

A new planthopper species *Dictyotenguna angusta* **sp. n.** is described and illustrated from Guangxi, China. The photographs of the adults of the species are presented.

## Introduction

The Oriental dictyopharid planthopper genus *Dictyotenguna* was established by Song & Liang (2012) for a single species, *Dictyotenguna choui* Song & Liang, from China. Here we describe and illustrate a second species of the genus from Guangxi Autonomous Region, Southern China.

## Material and methods

Dry specimens were used for the observation, description and illustration. Genital segments of the specimens examined were macerated in boiling solution of 10% NaOH and drawn from preparations in glycerin jelly under a Leica MZ12.5 stereomicroscope. Color pictures for adult habitus were obtained by a KEYENCE VHX-1000 system. Illustrations were scanned with Canon CanoScan LiDE 200 and imported into Adobe Photoshop CS6 for labeling and fig composition. Terminology of morphology, genital characters and measurements follow Yang and Yeh (1994).

The following abbreviations are used in the text, BL: body length (from apex of cephalic process to tip of fore wings); HL: head length (from apex of cephalic process to base of eyes); HW: head width (including eyes); FWL: forewing length.

Type specimens as well as material examined here are deposited in the Institute of Entomology, Guizhou University, Guiyang, China (GUGC).

## Taxonomy

### Dictyotenguna

Taxon classificationAnimaliaHemipteraDictyopharidae

Song & Liang

Dictyotenguna Song & Liang, 2012: 29.

#### Type species.

*Dictyotenguna choui* Song & Liang, 2012

#### Diagnosis.

For the relationship and diagnosis of *Dictyotenguna* see in Song and Liang (2012).

#### Distribution.

Oriental region.

### Dictyotenguna choui

Taxon classificationAnimaliaHemipteraDictyopharidae

Song & Liang

Dictyotenguna choui Song & Liang, 2012: 211.

#### Material examined.

CHINA: 1 ♂, Sichuan, Guangyuan, Micangshan, 21 August 2007, coll. Yubo Zhang. 1 ♂, Sichuan, Mianyang, Qianfoshan, 840 m, 13 August 2007, coll. Yubo Zhang.

#### Distribution.

China (Fujian, Guangxi and Sichuan).

### Dictyotenguna angusta sp. n.

Taxon classificationAnimaliaHemipteraDictyopharidae

http://zoobank.org/286B2C86-75D7-461C-8819-0B8846325E15

Figs 1–5 , 6 –16 , 17 –21

#### Description.

♂, BL: 14.6 mm HL: 1.8 mm HW: 1.7 mm FWL: 11.5 mm. ♀, BL: 17.3 mm HL: 2.0 HW: 1.8 mm FWL: 14.1 mm.

Body green. Carinae and veins of wings dark green. Frons between lateral intermediate carinae orange red. Rostrum blackish at extreme apex. Femora with a black marking at apex.

**Figures 1–5.**
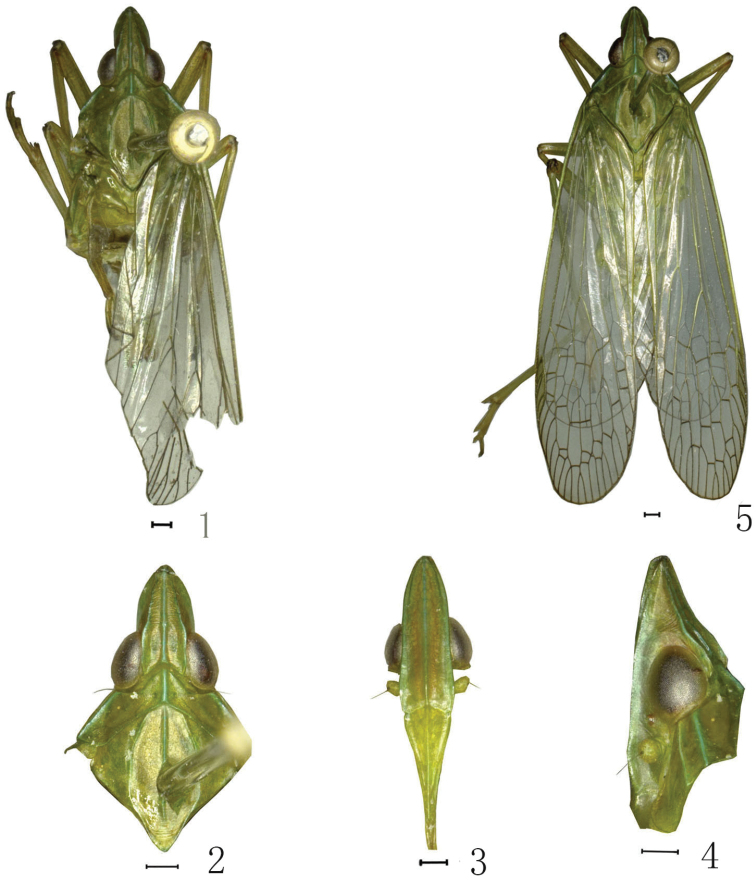
Habitus of *Dictyotenguna angusta* sp. n. **1** *Dictyotenguna angusta* sp. n., male, holotype **2** Same, head and thorax, dorsal view **3** Same, frons and clypeus, ventral view **4** Same, head and pronotum, lateral view **5** *Dictyotenguna angusta* sp. n., female, paratype. Scale bars: **1–5** = 0.5mm.

**Figures 6–16.**
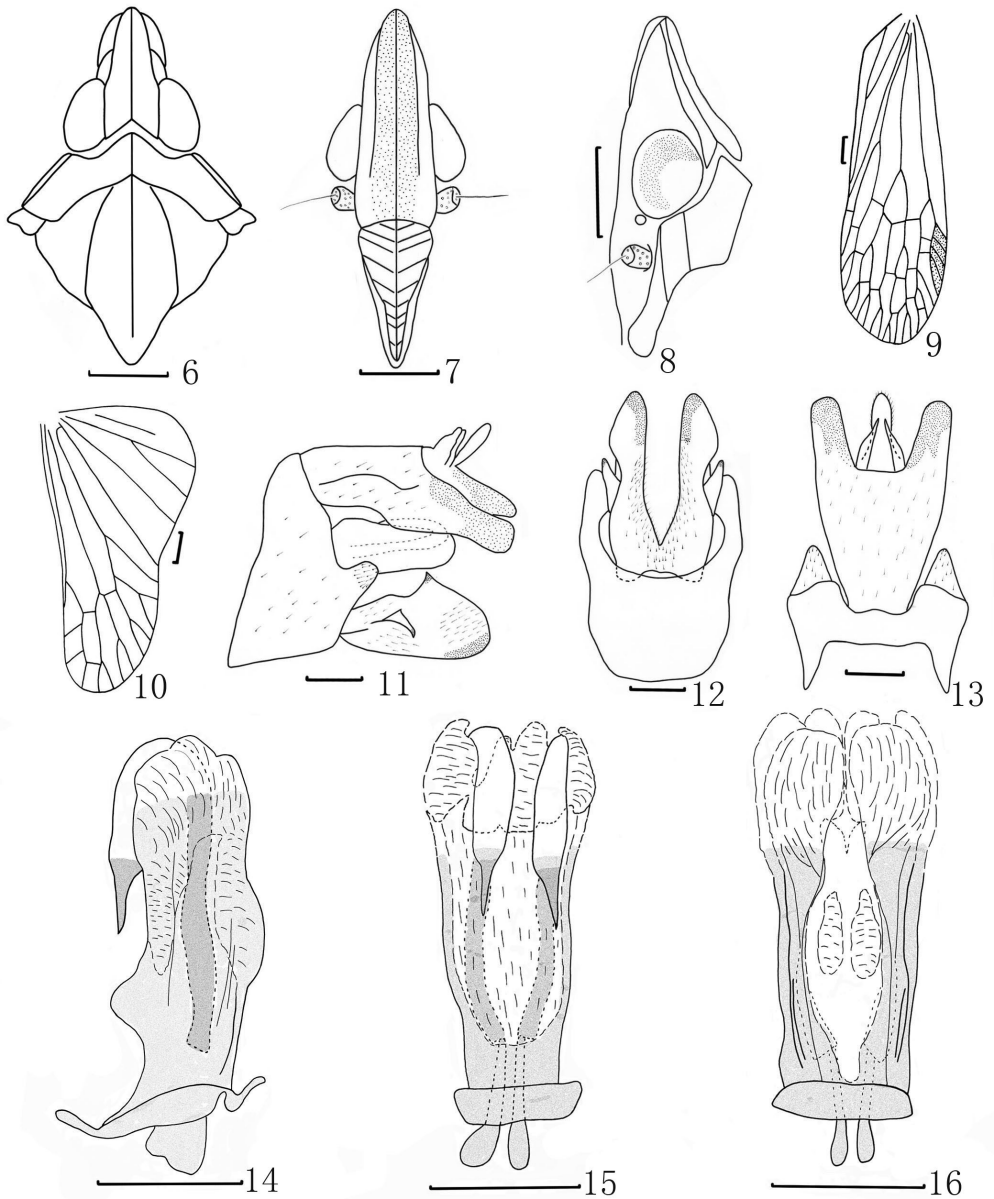
*Dictyotenguna angusta* sp. n. **6** Head and thorax, dorsal view **7** Frons and clypeus, ventral view **8** Head and pronotum, lateral view **9** Forewing **10** Hind Wing **11** Pygofer and anal tube, dorsal view**12** Pygofer and parameres, ventral view **13** Genitalia, lateral view **14** Aedeagus, lateral view **15** Aedeagus, dorsal view **16** Aedeagus, ventral view. Scale bars: **6–10** = 1 mm, **11–16** = 0.5mm.

Head (Figs 1, 2, 6) relatively short, shorter than pronotum and mesonotum combined, the ratio of length about 0.6:1. Vertex (Figs 1, 2, 4, 6, 8) relatively narrow, two times as wide as long between eyes, media carina conspicuous and strongly, lateral margins carinate sub-parallel at base, slightly sinuate in front of eyes, then gradually narrowing to arrowhead at apex. Frons (Figs 3, 7) nearly rectangle, length 2.5 times long than wide, lateral carinae reaching to the back of eyes. Pronotum (Figs 1–2, 6) distinctly shorter than mesonotum medially, with ratio about 0.2:1, disc broad with median carina distinct, lateral carina very faint. Mesonotum (Figs 1–2, 6) with median longitudinal carina diatinct, not reach to the apex, lateral carinae curverging at the front. Forewings (Figs 5, 9) with Sc+R, M and Cu all branched apically, stigma distinct, with 4 cells. Legs moderately elongate.

**Male genitalia.** Pygofer (Figs 11–13) with a large process on posterior margin, and the process with lots of setae; anterior margin relatively straight. Anal tube (Figs 11, 13) large and broad, apex U-shaped in dorsal view. Parameres (Figs 11, 12) large in lateral view, posterior margin with a dorsally directed black-tipped process, and with a ventrally directed process near sub-middle on outer upper edge. Aedeagus (Fig. 14) with a pair of processes extended dorsally. Phallobase (Figs 14–16) basally sclerotized and pigmented, with apical membranous lobes: dorsal apical lobes slender and connected (Fig. 15); ventral lobes composed of two parts: one pairs large on apex, the other one small and the base produced near middle part (Fig. 16).

**Female genitalia.** Anal tube (Fig. 18) round and large in dorsal view, with ratio of length to width at middle about 1:1. First valvulae (Fig. 19) sclerotized with 6 teeth of different sized in lateral view; second valvulae (Fig. 20) triangular, symmetrical in ventral view, connected at base and separated from 1/4 base; third valvulae (Fig. 21) with 2 sclerotized lobes, lateral lobe with 5 long spines at apex, and one of the five separate from others.

#### Type material.

Holotype ♂, China: Guangxi, Huaping, 900 m, 31 July 2007, coll. Pei Zhang. Paratype, 1 ♀, same data as holotype.

#### Etymology.

This new species is derived from the Greek word “*angusta*”, indicating that the apical lobes of phallobase slender.

#### Distribution.

China (Guangxi).

#### Remarks.

This species is similar to *Dictyotenguna choui* Song & Liang, but can be distinguished from the latter by aedeagus with a pair of processes extended anteriorly (Fig. 15) in dorsal view (processes extended to left and right sides in *D.Choui*), and pygofer with anterior margin quite straight(Fig. 11) in later view(anterior margin angular in *D.Choui*).

**Figures 17–21.**
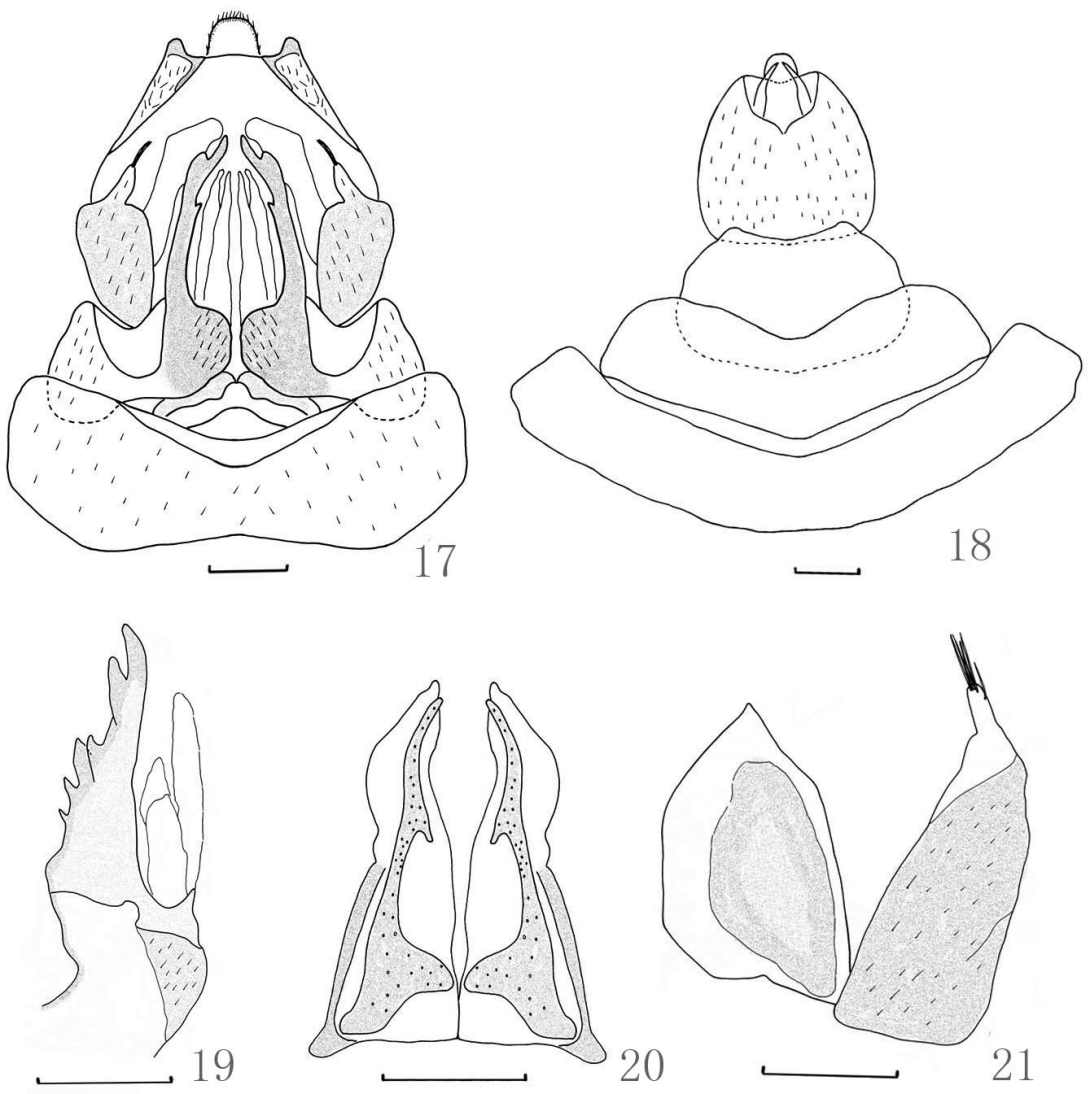
*Dictyotenguna angusta* sp. n. **17** Genitalia ventral view of female **18** Genitalia dorsal view of female **19** First valvulae (lateral view) **20** Second valvulae (ventral view) **21** Third valvulae (lateral view). Scale bars: **17–21** = 0.5mm.

## Supplementary Material

XML Treatment for Dictyotenguna

XML Treatment for Dictyotenguna choui

XML Treatment for Dictyotenguna angusta
